# Clinical trials for Wolfram syndrome neurodegeneration: Novel design, endpoints, and analysis models

**DOI:** 10.1371/journal.pone.0321598

**Published:** 2025-05-09

**Authors:** Guoqiao Wang, Zhaolong Adrian Li, Ling Chen, Heather Lugar, Tamara Hershey

**Affiliations:** 1 Department of Neurology, Washington University in St Louis School of Medicine, St Louis, Missouri, United States of America; 2 Division of Biostatistics, Washington University in St Louis School of Medicine, St Louis, Missouri, United States of America; 3 Department of Psychiatry, Washington University in St Louis School of Medicine, St Louis, Missouri, United States of America; 4 Department of Radiology, Washington University in St Louis School of Medicine, St Louis, Missouri, United States of America; University of Tartu, ESTONIA

## Abstract

**Objective:**

Wolfram syndrome, an ultra-rare condition, currently lacks effective treatment options. The rarity of this disease presents significant challenges in conducting clinical trials, particularly in achieving sufficient statistical power (e.g., 80%). The objective of this study is to propose a novel clinical trial design based on real-world data to reduce the sample size required for conducting clinical trials for Wolfram syndrome.

**Methods:**

We propose a novel clinical trial design with three key features aimed at reducing sample size and improve efficiency: (i) Pooling historical/external controls from a longitudinal observational study conducted by the Washington University Wolfram Research Clinic. (ii) Utilizing run-in data to estimate model parameters. (iii) Simultaneously tracking treatment effects in two endpoints using a multivariate proportional linear mixed effects model.

**Results:**

Comprehensive simulations were conducted based on real-world data obtained through the Wolfram syndrome longitudinal observational study. Our simulations demonstrate that this proposed design can substantially reduce sample size requirements. Specifically, with a bivariate endpoint and the inclusion of run-in data, a sample size of approximately 30 per group can achieve over 80% power, assuming the placebo progression rate remains consistent during both the run-in and randomized periods. In cases where the placebo progression rate varies, the sample size increases to approximately 50 per group.

**Conclusions:**

For rare diseases like Wolfram syndrome, leveraging existing resources such as historical/external controls and run-in data, along with evaluating comprehensive treatment effects using bivariate/multivariate endpoints, can significantly expedite the development of new drugs.

## 1 Introduction

Wolfram syndrome is a rare (estimated to affect 1 in 770,000–160,000 people), autosomal recessive, multisystem disease first defined in 1938 as the combination of childhood-onset insulin-dependent diabetes, optic nerve atrophy, diabetes insipidus, and hearing loss [1,2]. The major causative gene *WFS1* encodes for wolframin [3], an endoplasmic reticulum (ER) transmembrane glycoprotein involved in preventing cell death potentially through suppressing ER stress [4], regulating intracellular calcium homeostasis [5], and modulating mitochondrial function [6]. It is now understood that pathogenic mutations in *WFS1* can result in death or dysfunction of insulin-producing pancreatic β cells [3], causing insulin-dependent diabetes. *WFS1* mutations are also presumed to impact other organ systems [7], leading to a wide range of symptomatology. Several agents proposed to protect cells from ER stress-mediated apoptosis are being tested [5,8–12]; however, currently there are no approved treatments that alter Wolfram syndrome progression.

The identification of causative *WFS1* genetic mutations has allowed for a genetic rather than purely clinical diagnosis of Wolfram syndrome. Observations of genetically diagnosed Wolfram syndrome individuals indicate that the disease phenotype is indeed much more variable than classically understood, extending to loss of taste and smell [13–15], bladder and bowel dysfunction [16–18], gait and balance issues [19,20], and mental health disorders [13,21]. Wolfram syndrome is associated with early brain structural alterations such as reduced or stalled white matter development [22–25]. In addition, patterns of explicit neurodegeneration can be detected in gray matter regions over 2–3 years, regardless of age [23]. As these abnormalities progress and brain-mediated symptoms worsen, quality of life decreases and life-threatening conditions can develop (e.g., respiratory failure from brainstem atrophy) [26].

Identifying biomarkers of brain degeneration is thus a critical step towards designing efficient and effective clinical trials for candidate pharmaceutical agents or therapies. We have conducted the world’s largest and longest natural history study of early Wolfram syndrome, focusing on quantification of neurological features of the disease and their changes over time. Over the 13 years of the Washington University Wolfram Research Clinic (https://wolframsyndrome.wustl.edu/items/research-clinic/; ClinicalTrials.gov Identifier NCT03951298), we validated a standardized clinical severity rating scale for Wolfram syndrome (the Wolfram Unified Rating Scale, WURS) [27], described unexpectedly early neurological symptoms and neuropathological differences, and characterized the rate of change in these symptoms and in regional brain volumes. We found that visual acuity and the volume of the thalamus (based on magnetic resonance imaging [MRI]) deteriorate the most consistently and rapidly in our cohort [24,28]. This information has been used to justify outcome measures and predict power for safety [8] and clinical efficacy studies using standard designs (e.g., ClinicalTrials.gov Identifier: NCT03717909).

**Fig 1:**
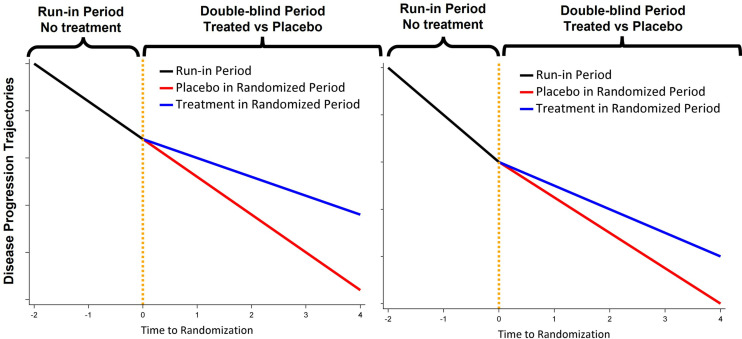
Demonstration of how clinical trials might use run-in data. Left: a trial design that uses the same disease progression rate for placebo during the run-in period and the randomized period; Right: a trial design that uses different disease progression rates for placebo during the run-in period and the randomized period.

One of the primary challenges for clinical trials in rare diseases such as Wolfram syndrome is enrolling an adequate number of participants to ensure sufficient statistical power. Here, we leverage our unique longitudinal natural history dataset to explore the added power obtained (and reduced sample sizes required) by using three different innovative design features. First, we consider the impact of including historical data from patients who were not treated in the clinical trial. This approach allows more participants to receive active treatment without compromising statistical power compared to the traditional 1:1 randomization trial [29,30]. For example, a trial with 2:1 randomization can be analyzed as if it had a 1:1 ratio by borrowing the same number of historical/external controls as the randomized placebo controls. Second, we consider how best to use data collected from Wolfram syndrome patients prior to their participation in a clinical trial (i.e., ‘run-in’ data [31,32]). Run-in data aids in estimating important model parameters such as the rate of change of the placebo group or the variances, thereby enhancing statistical power [31,32]. Depending on whether the disease progression remains relatively stable during a moderate follow-up duration (e.g., a total of 4–6 years), the disease progression rate for participants on placebo can be assumed to be the same or different during the run-in period and the randomized period (Fig 1).

Finally, a single primary endpoint may not fully capture the important effects of an intervention, particularly for diseases with diverse manifestations such as Wolfram syndrome. In such cases, the use of multiple primary endpoints presents an appealing solution [33]. The multiple endpoints can be modeled in several ways: as a multivariate endpoint [34], as a composite endpoint (e.g., average [30,35] or sum [36]), or by analyzing each endpoint individually in a sequential manner. The use of an integrated scale such as a composite endpoint [37,38] has occurred in Alzheimer’s disease trials and can be acceptable to the FDA [39]. However, a composite endpoint may impose challenges in interpretation and may inadvertently assign disproportionate weight to a particular endpoint upon averaging or summing [40]. Analyzing multiple endpoints sequentially requires careful control of type I error and can sometimes result in a loss of statistical power [41]. To address these limitations, we propose a direct modeling approach that considers multiple endpoints simultaneously using proportional mixed models for repeated measures [42]. This approach allows for the identification of a single proportional reduction in disease progression across multiple endpoints, effectively capturing the treatment effect across a range of measures.

In this paper, we present the impact of these three design features on the predicted power and required sample sizes in clinical trials of interventions that target neurodegeneration in Wolfram syndrome. By using our unique longitudinal dataset from a natural history study of Wolfram syndrome, we can empirically determine the design that optimally balances the power to detect slowing of neurodegeneration while minimizing the required sample size. Future clinical trials targeting neurodegeneration in Wolfram syndrome may then use this information to optimize their trial designs and justify their proposed sample sizes.

## 2 Methods

**Study Oversight:** This study does not involve human subjects and is based on simulated data using information obtained from de-identified group-level data. Therefore, it does not require IRB approval. Consequently, participant consent is not required for this study.

**Ethics approval and consent to participate do not apply to this study as it is primarily based on simulated data.**

### 2.1 Data

Participants with genetically confirmed *WFS1* mutations, and under the age of 30 at enrollment, were assessed annually at the Washington University Wolfram Syndrome Research Clinic[23]. Briefly, at each annual visit participants were evaluated by a pediatric ophthalmologist or optometrist who measured best corrected visual acuity by Snellen optotype [28]. In the following analyses, we used the logarithm of the minimum angle of resolution (logMAR) value for best-corrected visual acuity with both eyes open; higher logMAR value indicates worse visual acuity. Participants also underwent magnetic resonance imaging where a T1-weighted high-resolution magnetization-prepared rapid gradient-echo (MPRAGE) sequence was acquired. Scans from each annual visit were processed longitudinally using the semi-automatic segmentation program FreeSurfer v5.3 [43], and regional brain volumes were extracted and corrected for estimated total intracranial volume (eTIV) [44]. Data acquired between 2010 and 2017 were included in these analyses.

### 2.2 Models

#### 2.2.1 Univariate linear mixed effects model.

The univariate linear mixed effects (LME) model that estimates a linear disease progression rate can be written as [42,45]:


$$y_{ijk}=\left(\mu_{0}+u_{0i}\right)+\left(\beta_{k}+u_{1i}\right)t_{ij}+\varepsilon_{ij}$$ (1)

where $y_{ijk}$ denotes the longitudinal assessments for subject i at time j for group k, i=1,2,…,n, $j=0,1,\ldots ,n_{i}$, with j=0 representing the baseline visit, and k=1,2 representing the placebo group and the treatment group, respectively; $\mu_{0}$ is the baseline intercept, $\beta_{k}$ is the disease progression rate for group k where time $t_{ij}$ represents subject-specific assessment time; $u_{0i},u_{1i}$ are the random effects for the intercept and the progression rate (i.e., slope) and are assumed to follow the same bivariate normal distribution for both groups: $\left(\begin{array}{c} u_{0i}\\ u_{1i} \end{array}\right)\sim N\left(0,\left[\begin{array}{cc} \sigma_{u_{0i}}^{2} & \sigma_{u_{0i}u_{1i}}\\ \sigma_{u_{0i}u_{1i}} & \sigma_{u_{1i}}^{2} \end{array}\right]\right)$; the within-subject error is assumed to follow the same normal distribution for both groups $\varepsilon_{ij}\sim N\left(0,\sigma_{e}^{2}\right)$.

#### 2.2.2 Treatment effect represented by difference in disease progression rates or proportional/percent reduction.

When analyzing the primary endpoint using model (1), the treatment effect is commonly expressed as the difference between the annual disease progression rates, i.e., $\beta_{1}-\beta_{2}$ for the two subject groups. An alternative approach is to model the treatment effect as the proportional/percent reduction in the disease progression rate relative to the placebo rate, as described below [29,42]:


$$y_{ijk}=\left(\mu_{0}+u_{0i}\right)+\left(\left(1-\theta_{T}I\left(k=2\right)\right)\beta_{1}+u_{1i}\right)t_{ij}+\varepsilon_{ij}$$ (2)

where $\theta_{T}$ is the proportional/percentage reduction treatment effect; I is the indicator function with I(k=2)=1 and I(k=1)=0; $\beta_{1}$ is the disease progression rate of the placebo group, and all the other parameters have the same meanings as those in model (1).

#### 2.2.3 Multivariate proportional linear mixed effects model.

Utilizing a proportional/percent reduction to represent the treatment effect offers several advantages: 1) enhances the interpretability of the treatment effect compared to solely focusing on the difference in slope of the absolute values; 2) enables the totality evaluation of the overall treatment effect across multiple endpoints; and 3) allows for a reduction in sample size by simultaneously modeling multivariate endpoints. Such multivariate endpoints can be modeled using a multivariate proportional mixed effects model as below, which accounts for the correlation among different endpoints through the use of random effects [42]:


$$y_{ijck}=\left(\mu_{0c}+u_{0ic}\right)+\left(\left(1-\theta_{T}I\left(k=2\right)\right)\beta_{1c}+\;\;\;\;u_{1ic}\;\;\;\;\right)t_{ij}+\varepsilon_{ijc}$$ (3)

Where, i represents the subject; j represents the visit/time where j=0 at baseline; c is the index for the endpoint included in the multivariate endpoint; k=1,2 represents the placebo group and the treatment group, respectively; I is the indicator function with I(k=2)=1 and I(k=1)=0; $\mu_{0c}$ and $\beta_{1c}$ represent the baseline intercept and the progression rate of the placebo group for endpoint c, respectively; $u_{0ic},u_{1ic}$ are the random effects for the intercept and the progression rate (slope) for subject i endpoint c, and are assumed to follow a multivariate normal distribution $\text{N}\left(0_{2c},\;\;\;\;{\;\;\Sigma \;\;}_{2c\times 2c}\right)$ (e.g., 4 by 4 unstructured covariance matrix if there are 2 endpoints); $\theta_{T}$ is the treatment effect represented as a proportional/percentage reduction of the placebo disease progression; and $\varepsilon_{ijc}$ is the within-subject residual and has different variances for different endpoints.

## 3 Results

Characteristics of participants in the Wolfram syndrome longitudinal observational study have been previously described [23]. In the current study, data from a total of 37 patients genetically diagnosed with Wolfram syndrome were included. Among them, 21 individuals (57%) were female. The mean (SD) baseline age of the participants was 13.3 (5.5) years, and the mean (SD) follow-up duration was 2.9 (1.2) years.

### 3.1 Estimated annual disease progression rates and the associated variance/covariance

A bivariate linear mixed-effects model was employed to estimate the annual progression rates and the associated variance/covariance components for both visual acuity and thalamus volume simultaneously in participants with Wolfram syndrome. These two biomarkers were found to deteriorate the most consistently and rapidly in previous longitudinal observational data [24,28], suggesting usefulness as disease progression endpoints. To facilitate model convergence, thalamus volumes were normalized to z-scores using the baseline mean (SD) of healthy controls. This adjustment made the range of normalized thalamus volume and visual acuity more compatible. Estimates are presented in Table 1 and Table 2. For interpretability, the estimated intercept for visual acuity of 0.540 logMAR corresponded to about 20/69 ft (6/21 m; 3^rd^ line) on the Snellen scale, and the estimated slope of 0.062 logMAR/y translated to approximately 7.4 years to deteriorate to 20/200 ft (6/60 m; 1^st^ line).

**Table 1:** Estimated annual disease progression rates and 95% CI for each endpoint.

Parameter	Estimate	SE	P-value	95% Confidence Limits	
Intercept of Visual Acuity (logMAR)	0.540	0.069	<.0001	0.399	0.681
Slope of Visual Acuity(LogMAR/year)	0.062	0.013	<.0001	0.035	0.089
Intercept of Thalamus Volume (z-score)	-1.116	0.165	<.0001	-1.451	-0.781
Slope of Thalamus Volume (z-score/year)	-0.139	0.023	<.0001	-0.185	-0.093

**Table 2:** Estimated variance/covariance components for each endpoint.

	Intercept of Visual Acuity	Slope of Visual Acuity	Intercept of Thalamus Volume	Slope of Thalamus Volume
Intercept of Visual Acuity	0.174	0.020	-0.178	-0.020
Slope of Visual Acuity	0.020	0.004	-0.042	-0.002
Intercept of Thalamus Volume	-0.178	-0.042	0.811	0.025
Slope of Thalamus Volume	-0.020	-0.002	0.025	0.007
Residual Variance of Visual Acuity	0.025			
Residual Variance of Thalamus Volume	0.005			

### 3.2 Evaluation of model performance by study designs and endpoints

To evaluate model performance for each study design (i.e., historical controls, run-in data) and each type of endpoints (i.e., univariate, multivariate), we conducted simulations for various scenarios. The simulations were configured as follows:

- Individual baseline values and annual progression rates for visual acuity and thalamus volume were generated using a multivariate normal distribution with the mean and variance/covariance components specified in **Table 1** and **Table 2**.
- Individual longitudinal data were simulated by combining the individual baseline values, annual progression rates, and within-subject error, according to model 3.
- Treatment effects were introduced as a proportional reduction in the placebo rate of progression at various levels: 0% (Type I error), 30%, and 40%.
- Time was set to 0 at baseline, negative before baseline (i.e., the run-in period), and positive after baseline.
- The run-in period lasted for 2 years, and the treatment period lasted for 3 years.
- Assessments were scheduled every 6 months.
- The per-visit dropout rate was set at 4.5%.
- The randomization ratio was 2:1 with pooled historical/external controls to make the power estimation based on a randomization ratio of 1:1, the sample sizes range from 30 per group to 120 per group.
- For each model, 1000 data sets were simulated, allowing for estimation of power with precision up to three decimal places [46].
- Type I error and power were calculated as the proportion of the 1000 simulated trials per scenario that yielded P-values < 0.05.
- Disease progression rates during both the run-in and randomized periods are the same vs different (slower progression during the randomized period due to the placebo and/or trial effect).

#### 3.2.1 Type I error/power estimation with vs. without run-in data for a single primary endpoint (Visual acuity).

**Fig 2:**
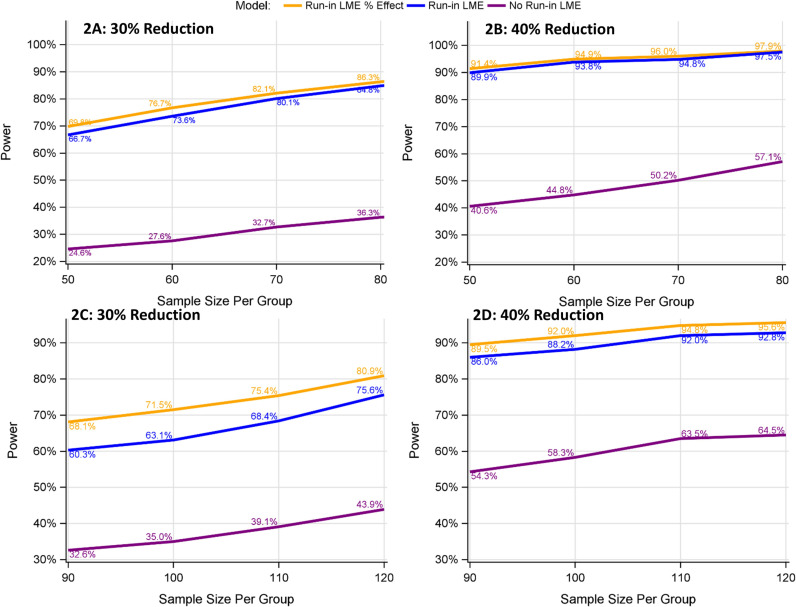
Power comparison between models with vs. without run-in data for the univariate endpoint of visual acuity. **A** and **B**: The disease progression rate for placebo participants is assumed to be the same throughout both the run-in and randomized periods (as in left panel in Fig 1). **C** and **D**: The disease progression rate for placebo participants is assumed to be slower in the randomized than the run-in period (as in right panel in Fig 1). 30%, 40% reduction: 30%, 40% reduction in the disease progression relative to the placebo group. No Run-in LME (purple lines): without run-in data analyzed using LME model 1. Run-in LME (blue lines): with run-in data analyzed using LME model 1. Run-in LME % Effect: (yellow lines) with run-in data analyzed using LME model 2.

Fig 2 illustrates the power of various design and analysis models. The inclusion of run-in data significantly enhances power compared to trials without run-in data, regardless of whether the placebo progression rates remain the same or not throughout both run-in and randomized periods. Nevertheless, when the placebo progression rates are the same during run-in and randomized periods (Fig 2A and 2B), utilization of run-in data results in a greater increase in power compared to scenarios where the placebo progression rates change (Fig 2C and 2D). Estimating the treatment effect as a percent reduction demonstrates higher power compared to estimating it as the difference in disease progression rates. Moreover, such gain in power is more pronounced when the disease progression rates for placebo participants differ between the run-in and randomized periods. The type I error for all designs/models generally remains within 2% of the nominal 5% level (S1 Fig).

#### 3.2.2 Type I error/power estimation for bivariate endpoint (visual acuity and thalamus volume).

**Fig 3:**
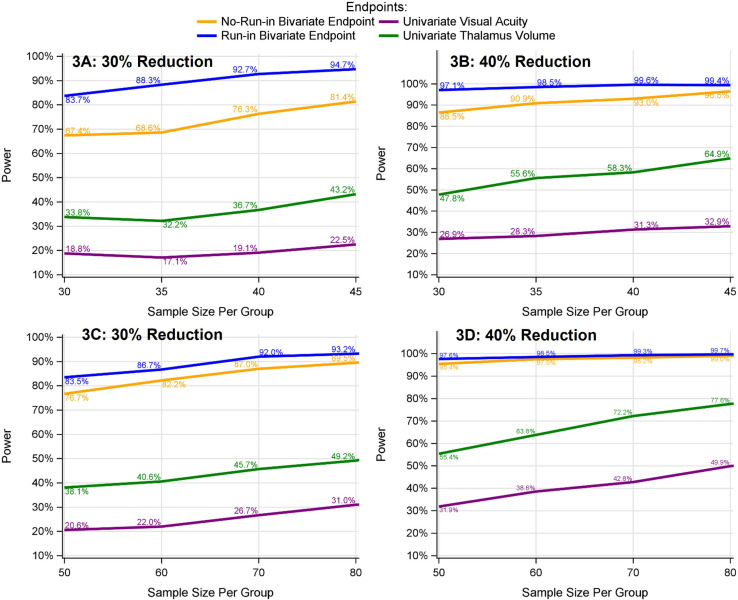
Power comparison between models with bivariate vs. univariate endpoints and with vs. without run-in data. **A** and **B**: The disease progression rate for placebo participants is assumed to be the same throughout both run-in and randomized periods (as in left panel in Fig 1). **C** and **D**: The disease progression rate for placebo participants is assumed to be slower during the randomized than run-in period (as in right panel in Fig 1). 30%, 40% reduction: 30%, 40% reduction in the disease progression relative to the placebo group. Univariate Visual Acuity: visual acuity analyzed by LME model 1. Univariate Thalamus Volume: thalamus volume analyzed by LME model 1; No-Run-in Bivariate Endpoint: bivariate endpoint of visual acuity and thalamus volume analyzed by model 3 without run-in data; Run-in Bivariate Endpoint: bivariate endpoint of visual acuity and thalamus volume analyzed by model 3 with run-in data.

Fig 3 presents an illustration of power associated with bivariate endpoints, with or without run-in data. Consistent with the simulations conducted for the univariate endpoint models, the inclusion of run-in data enhances power considerably. Further, the bivariate endpoint provides a significant gain in power, effectively doubling or even tripling the power observed with the univariate endpoint. Overall, the type I error for all endpoints/designs typically stays within 2% of the expected 5% level (S1 Fig).

## 4 Discussion

Our results demonstrate the range of power that can be obtained in clinical trials targeting neurodegeneration in Wolfram syndrome using practical and data-driven approaches. By leveraging our unique existing natural history dataset, we demonstrate the considerable potential gain in power achieved by using run-in and historical/external control data and by choosing a bivariate primary endpoint, assuming a 3-year follow-up with 6-month assessment intervals. After taking into account the assumptions and caveats associated with these models, investigators will be able to use this information and maximize existing resources to guide and standardize clinical trial protocols going forward, leading to more efficient trial designs for this ultra-rare disease.

The three features of the proposed design that contributed to increased power were the borrowing of historical/external controls, use of run-in data, and a bivariate primary endpoint of visual acuity and thalamus volume. Each of these design features boosted our power estimates, although there are some additional issues that need to be discussed. First, by leveraging historical data from our natural history study, we can employ an unequal randomization ratio, such as 2:1 with two times the number of patients on the active treatment. Our simulations assumed that we could borrow enough historical/external controls so that the efficacy analysis would have a 1:1 randomization ratio. In a real-world trial, however, the availability of historical/external controls will vary. Second, although each individual had 2 years of run-in data in the simulation, it is not necessary that the run-in data have equal duration. Any amount of run-in data with various durations can help [31]. In any trial that includes run-in data, it is recommended to conduct tailored simulations to evaluate the specific power gain from these run-in data. Unlike run-in data, which originate from participants in the trial, external controls are often collected from patients not directly involved in the ongoing trial. To ensure their appropriateness and comparability with concurrent controls, several measures should be implemented: (i) Apply the same inclusion and exclusion criteria to both the external control group and the concurrent control group to maintain a similar baseline population. (ii) Use techniques such as propensity score matching to align external controls with concurrent controls based on demographic, clinical, and other relevant baseline characteristics. (iii) Perform adjustments during the analysis to account for any residual differences in baseline characteristics between external and concurrent controls. (iv) Select external controls from studies or datasets with comparable conditions, including similar disease stages, treatment environments, and diagnostic criteria. These steps ensure that the target population for treatment effect estimation is consistent between trial participants and external controls, thereby reducing the risk of bias and enhancing the validity of the study results.

Finally, our simulations indicated that relying on a single endpoint is not ideal for such a rare disease with a wide spectrum of symptomatology. A bivariate endpoint (visual acuity and thalamus volume) not only significantly improved statistical power, but also would provide a more comprehensive evaluation of treatment effects across the disease spectrum without compromising type I error control. However, the bivariate endpoint method assumes that the proportional treatment effect is approximately the same across endpoints. Although this assumption might seem strong, it has been implicitly used in various composite endpoints [29,30,47]. When the proportional treatment effect differs between endpoints, the model will estimate an average treatment effect across all endpoints, similar to the use of a composite score of averaging multiple endpoints [29,30,47]. We recommend conducting a sensitivity bivariate analysis to estimate the proportional treatment effect for each endpoint separately. By comparing the average treatment effect to endpoint-specific treatment effects, a comprehensive evaluation of the treatment effect can be achieved. In this study, we compare various scenarios based on the power differences for a given sample size. Alternatively, we can quantify the sample size savings required to achieve 80% power. For example, in Fig 2B, our simulations show that the traditional LME model requires a sample size of 140 per group to achieve approximately 81% power. In contrast, the run-in LME % Effect model achieves about 80% power with only 40 participants per group, resulting in a savings of about 100 participants per group. Researchers can adapt the provided sample SAS code to evaluate sample size savings for other scenarios in a similar manner.

While not directly addressed in our simulations, it may be beneficial for the field to consider a platform trial with a master protocol for early Wolfram syndrome. A platform trial is a method for evaluating multiple targeted therapies within a single disease setting, using a perpetual framework where therapies can enter or exit the platform based on predefined decision algorithms [48]. Building platform trials with master protocols for Wolfram syndrome will enable more efficient utilization of resources and accelerate the pace of drug discovery. Such trials facilitate the establishment of a trial network infrastructure that encourages extensive collaboration among researchers in the field of rare diseases [48]. Currently, a group of investigators across Europe are conducting a multi-site clinical trial (ClinicalTrials.gov Identifier: NCT03717909) with a master protocol. We would advocate for a similar approach within the US, ideally calibrating measures across sites, clinics and regions.

Our study has several limitations. First, our natural history data were obtained from a non-random group of participants. Enrolled participants were generally young, in an early disease state, and able to travel to St. Louis for annual several day-long visits. In addition, they were able to perform visual acuity tests (i.e., were not already blind) and did not have any contraindications for MRI scans (e.g., no cochlear implants, bladder stimulators, etc.). As a result, older, more impaired participants were not included in the study. However, the younger and less impaired individuals are the ones that presumably would benefit the most from slowing neurodegeneration and avoiding irreversible consequences of Wolfram syndrome. Second, there may be other, unexplored measures of neurodegeneration that could be more precise or more easily collected than MRI-based regional brain volumes. For example, fluid biomarkers are becoming more available and acceptable in clinical trials for other neurodegenerative conditions [37,49] and may have promise in Wolfram syndrome [50]. Third, the proposed multivariate proportional linear mixed-effects model assumes linearity in the disease progression trajectory to estimate an overall comparison between treatment and placebo groups. However, when linearity is a concern, alternative methods can be employed. For example, non-parametric approaches like the mixed model for repeated measures (MMRM) do not rely on a linearity assumption [47]. Additionally, parametric methods, such as models using spline-based time variables, can capture more complex, nonlinear progression patterns [38]. These methods can also incorporate a proportional treatment effect framework, allowing multiple endpoints to be combined, which may enhance statistical power compared to testing single endpoints independently [29,42,51,52]. Lastly, our study aims to enhance the feasibility of clinical trials for rare diseases by reducing sample size requirements through the use of alternative bivariate endpoints and proportional linear mixed-effects models. However, the selection of specific endpoints for inclusion in the bivariate endpoint, as well as the assumptions underlying the model, should be carefully evaluated using available data, including historical datasets or results from phase 2 trials, before implementation in a phase 3 trial.

In this paper, we propose a novel paradigm for conducting clinical trials in Wolfram syndrome, focusing on optimizing available resources and statistical approaches to enhance the feasibility of trials in this rare disease. The use of historical/external controls, run-in data, and a bivariate endpoint can greatly reduce the sample size required, thus facilitating the progress towards identifying truly effective drugs for this rare disease.

## Supporting information

S1 Fig Type I error by sample size for different endpoints/models. A: Single primary endpoint, placebo progression rates remain the same during both the run-in and randomized periods; B: Single primary endpoint, placebo progression rates are different during the run-in and randomized periods; C: Placebo progression rates remain the same during both the run-in and randomized periods; D: Placebo progression rates are different during the run-in and randomized periods.
(DOCX)

## List of Abbreviations:

ER: endoplasmic reticulum
WURS: Wolfram Unified Rating Scale
MRI: magnetic resonance imaging
logMAR: logarithm of the minimum angle of resolution
eTIV: estimated total intracranial volume
LME: linear mixed effects
