# Supplementary material for: Clinical trials for Wolfram syndrome neurodegeneration: Novel design, endpoints, and analysis models
Source: PLoS One. 2025 May 9;20(5):e0321598. doi: 10.1371/journal.pone.0321598 (PMC12064034; doi:10.1371/journal.pone.0321598)
Supplement: S1 Fig — A: Single primary endpoint, placebo progression rates remain the same during both the run-in and randomized periods; B: Single primary endpoint, placebo progression rates are different during the run-in and randomized periods; C: Placebo progression rates remain the same during both the run-in and randomized periods; D: Placebo progression rates are different during the run-in and randomized periods. (DOCX) [file pone.0321598.s001.docx]

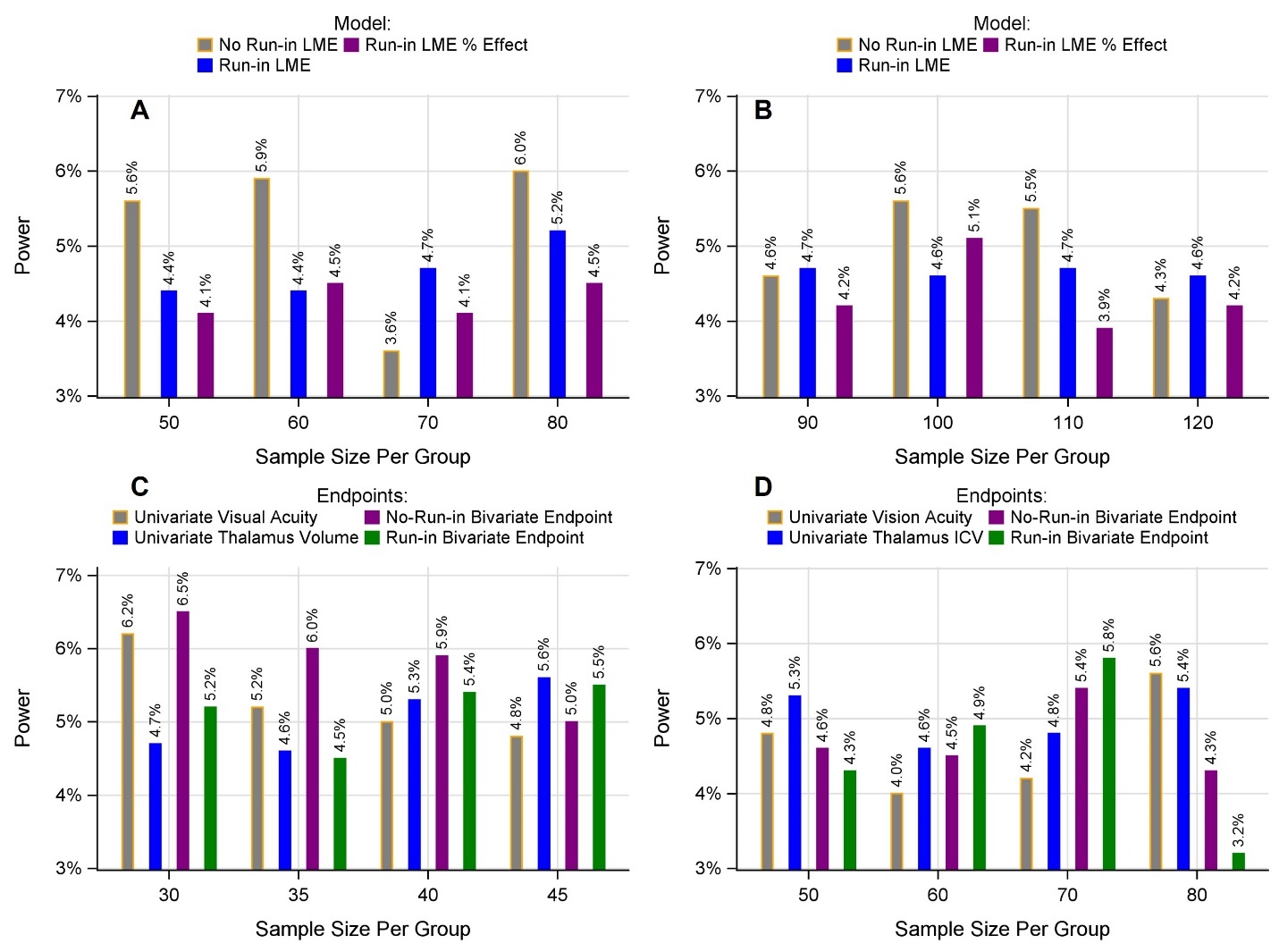


**Supporting Caption:** Supplemental Figure 1 illustrates the Type I error rates as a function of sample size for different endpoints and models.

**Supplemental Figure 1**: Type I error by sample size for different endpoints/models. A: Single primary endpoint, placebo progression rates remain the same during both the run-in and randomized periods; B: Single primary endpoint, placebo progression rates are different during the run-in and randomized periods; C: Placebo progression rates remain the same during both the run-in and randomized periods; D: Placebo progression rates are different during the run-in and randomized periods.

**Sample Code**

*Questions: guoqiao.wang@gmail.com or guoqiao@wustl.edu

*Simulation Parameters were estimated from the observational study;

**data** cov(type=COV) ;

input _TYPE_ $ **1**-**4** _NAME_ $ **5**-**6** S1 S2 S3 S4;

datalines ;

COV S1 0.174 0.020 -0.178 -0.020

COV S2 0.020 0.004 -0.042 -0.002

COV S3 -0.178 -0.042 0.811 0.025

COV S4 -0.020 -0.002 0.025 0.007

MEAN 0.5398 0 -1.1158 0

;**run**;

*Simulate concurrent placebo participants;

*total sample size 60/arm, 30 concurrent placebo + 30 external controls;

*First, simulate 30 concurrent placebos;

**proc** **simnorm** data=cov outsim=PBO_outcome numreal =**30**; var s1-s4;**run**;

**data** PBO_outcome;set PBO_outcome;

group='P';

rename s1=int0 s2=ind_rc0 s3=int1 s4=ind_rc1;

**run**;

**data** p01_wide;set PBO_outcome;

id=_n_;

outcome=**1**;

z_01=int0-(**0.062**+ind_rc0)***0.5**+rand('normal',**0**,**0.0707**);

z_02=int0-(**0.062**+ind_rc0)***1**+rand('normal',**0**,**0.0707**);

z_03=int0-(**0.062**+ind_rc0)***1.5**+rand('normal',**0**,**0.0707**);

z_04=int0-(**0.062**+ind_rc0)***2**+rand('normal',**0**,**0.0707**);

z_0=int0+rand('normal',**0**,**0.0707**);

*Assume that the delcine slows down slightly after enrolled into the clinical trial: so called trial effect;

z_1=int0+(**0.062**-**0.005**+ind_rc0)***0.5**+rand('normal',**0**,**0.0707**);

z_2=int0+(**0.062**-**0.005**+ind_rc0)***1**+rand('normal',**0**,**0.0707**);

z_3=int0+(**0.062**-**0.005**+ind_rc0)***1.5**+rand('normal',**0**,**0.0707**);

z_4=int0+(**0.062**-**0.005**+ind_rc0)***2**+rand('normal',**0**,**0.0707**);

z_5=int0+(**0.062**-**0.005**+ind_rc0)***2.5**+rand('normal',**0**,**0.0707**);

z_6=int0+(**0.062**-**0.005**+ind_rc0)***3**+rand('normal',**0**,**0.0707**);

keep id outcome z_01-z_04 z_0-z_6 group;

**run**;

**data** p02_wide;set PBO_outcome;

id=_n_;

outcome=**2**;

z_01=int1-(-**0.1389**+ind_rc1)***0.5**+rand('normal',**0**,**0.1590**);

z_02=int1-(-**0.1389**+ind_rc1)***1**+rand('normal',**0**,**0.1590**);

z_03=int1-(-**0.1389**+ind_rc1)***1.5**+rand('normal',**0**,**0.1590**);

z_04=int1-(-**0.1389**+ind_rc1)***2**+rand('normal',**0**,**0.1590**);

z_0=int1+rand('normal',**0**,**0.1590**);

*Assume that the delcine slows down slightly after enrolled into the clinical trial: so called trial effect;

z_1=int1+(-**0.1389**+**0.02**+ind_rc1)***0.5**+rand('normal',**0**,**0.1590**);

z_2=int1+(-**0.1389**+**0.02**+ind_rc1)***1**+rand('normal',**0**,**0.1590**);

z_3=int1+(-**0.1389**+**0.02**+ind_rc1)***1.5**+rand('normal',**0**,**0.1590**);

z_4=int1+(-**0.1389**+**0.02**+ind_rc1)***2**+rand('normal',**0**,**0.1590**);

z_5=int1+(-**0.1389**+**0.02**+ind_rc1)***2.5**+rand('normal',**0**,**0.1590**);

z_6=int1+(-**0.1389**+**0.02**+ind_rc1)***3**+rand('normal',**0**,**0.1590**);

keep id outcome z_01-z_04 z_0-z_6 group;

**run**;

*Simulate 30 external control;

*Both concurrent placebo and external controls are assumed to follow the same distribution.

This assumption can be modified to investigate different scenarios;

**proc** **simnorm** data=cov outsim=ec_outcome numreal =**30**; var s1-s4;**run**;

**data** ec_outcome;set ec_outcome;

group='P';

rename s1=int0 s2=ind_rc0 s3=int1 s4=ind_rc1;

**run**;

**data** p03_wide;set ec_outcome;

id=_n_+**30**;

outcome=**1**;

z_01=int0-(**0.062**+ind_rc0)***0.5**+rand('normal',**0**,**0.0707**);

z_02=int0-(**0.062**+ind_rc0)***1**+rand('normal',**0**,**0.0707**);

z_03=int0-(**0.062**+ind_rc0)***1.5**+rand('normal',**0**,**0.0707**);

z_04=int0-(**0.062**+ind_rc0)***2**+rand('normal',**0**,**0.0707**);

z_0=int0+rand('normal',**0**,**0.0707**);

*Assume that the delcine slows down slightly after enrolled into the clinical trial: so called trial effect;

z_1=int0+(**0.062**-**0.005**+ind_rc0)***0.5**+rand('normal',**0**,**0.0707**);

z_2=int0+(**0.062**-**0.005**+ind_rc0)***1**+rand('normal',**0**,**0.0707**);

z_3=int0+(**0.062**-**0.005**+ind_rc0)***1.5**+rand('normal',**0**,**0.0707**);

z_4=int0+(**0.062**-**0.005**+ind_rc0)***2**+rand('normal',**0**,**0.0707**);

z_5=int0+(**0.062**-**0.005**+ind_rc0)***2.5**+rand('normal',**0**,**0.0707**);

z_6=int0+(**0.062**-**0.005**+ind_rc0)***3**+rand('normal',**0**,**0.0707**);

keep id outcome z_01-z_04 z_0-z_6 group;

**run**;

**data** p04_wide;set ec_outcome;

id=_n_+**30**;

outcome=**2**;

z_01=int1-(-**0.1389**+ind_rc1)***0.5**+rand('normal',**0**,**0.1590**);

z_02=int1-(-**0.1389**+ind_rc1)***1**+rand('normal',**0**,**0.1590**);

z_03=int1-(-**0.1389**+ind_rc1)***1.5**+rand('normal',**0**,**0.1590**);

z_04=int1-(-**0.1389**+ind_rc1)***2**+rand('normal',**0**,**0.1590**);

z_0=int1+rand('normal',**0**,**0.1590**);

*Assume that the delcine slows down slightly after enrolled into the clinical trial: so called trial effect;

z_1=int1+(-**0.1389**+**0.02**+ind_rc1)***0.5**+rand('normal',**0**,**0.1590**);

z_2=int1+(-**0.1389**+**0.02**+ind_rc1)***1**+rand('normal',**0**,**0.1590**);

z_3=int1+(-**0.1389**+**0.02**+ind_rc1)***1.5**+rand('normal',**0**,**0.1590**);

z_4=int1+(-**0.1389**+**0.02**+ind_rc1)***2**+rand('normal',**0**,**0.1590**);

z_5=int1+(-**0.1389**+**0.02**+ind_rc1)***2.5**+rand('normal',**0**,**0.1590**);

z_6=int1+(-**0.1389**+**0.02**+ind_rc1)***3**+rand('normal',**0**,**0.1590**);

keep id outcome z_01-z_04 z_0-z_6 group;

**run**;

*simulate the 60 participants in the treatment group;

**proc** **simnorm** data=cov outsim=Treatment_outcome numreal =**60**; var s1-s4;**run**;

**data** Treatment_outcome;set Treatment_outcome;

group='T';

rename s1=int0 s2=ind_rc0 s3=int1 s4=ind_rc1;

**run**;

**data** p11_wide;set Treatment_outcome;

id=_n_+**60**;

outcome=**1**;

z_01=int0-(**0.062**+ind_rc0)***0.5**+rand('normal',**0**,**0.0707**);

z_02=int0-(**0.062**+ind_rc0)***1**+rand('normal',**0**,**0.0707**);

z_03=int0-(**0.062**+ind_rc0)***1.5**+rand('normal',**0**,**0.0707**);

z_04=int0-(**0.062**+ind_rc0)***2**+rand('normal',**0**,**0.0707**);

z_0=int0+rand('normal',**0**,**0.0707**);

z_1=int0+((**0.062**-**0.005**)*(**1**-**0.3**)+ind_rc0)***0.5**+rand('normal',**0**,**0.0707**);

z_2=int0+((**0.062**-**0.005**)*(**1**-**0.3**)+ind_rc0)***1**+rand('normal',**0**,**0.0707**);

z_3=int0+((**0.062**-**0.005**)*(**1**-**0.3**)+ind_rc0)***1.5**+rand('normal',**0**,**0.0707**);

z_4=int0+((**0.062**-**0.005**)*(**1**-**0.3**)+ind_rc0)***2**+rand('normal',**0**,**0.0707**);

z_5=int0+((**0.062**-**0.005**)*(**1**-**0.3**)+ind_rc0)***2.5**+rand('normal',**0**,**0.0707**);

z_6=int0+((**0.062**-**0.005**)*(**1**-**0.3**)+ind_rc0)***3**+rand('normal',**0**,**0.0707**);

keep id outcome z_01-z_04 z_0-z_6 group;

**run**;

**data** p12_wide;set Treatment_outcome;

id=_n_+**60**;

outcome=**2**;

z_01=int1-(-**0.1389**+ind_rc1)***0.5**+rand('normal',**0**,**0.1590**);

z_02=int1-(-**0.1389**+ind_rc1)***1**+rand('normal',**0**,**0.1590**);

z_03=int1-(-**0.1389**+ind_rc1)***1.5**+rand('normal',**0**,**0.1590**);

z_04=int1-(-**0.1389**+ind_rc1)***2**+rand('normal',**0**,**0.1590**);

z_0=int1+rand('normal',**0**,**0.1590**);

z_1=int1+((-**0.1389**+**0.02**)*(**1**-**0.3**)+ind_rc1)***0.5**+rand('normal',**0**,**0.1590**);

z_2=int1+((-**0.1389**+**0.02**)*(**1**-**0.3**)+ind_rc1)***1**+rand('normal',**0**,**0.1590**);

z_3=int1+((-**0.1389**+**0.02**)*(**1**-**0.3**)+ind_rc1)***1.5**+rand('normal',**0**,**0.1590**);

z_4=int1+((-**0.1389**+**0.02**)*(**1**-**0.3**)+ind_rc1)***2**+rand('normal',**0**,**0.1590**);

z_5=int1+((-**0.1389**+**0.02**)*(**1**-**0.3**)+ind_rc1)***2.5**+rand('normal',**0**,**0.1590**);

z_6=int1+((-**0.1389**+**0.02**)*(**1**-**0.3**)+ind_rc1)***3**+rand('normal',**0**,**0.1590**);

keep id outcome z_01-z_04 z_0-z_6 group;

**run**;

**data** _4;

set p01_wide p02_wide p03_wide p04_wide p11_wide p12_wide;

**run**;

**data** _4;set _4;

array miss{*} miss1-miss6;

do i=**1** to **6**;

miss{i}=rand('uniform',**0**,**1**);

end;

drop i;

**run**;

*Dropout rate can be varied: 4.5% per visit in our simualtion;

**data** _4;set _4;

array miss{*} miss1-miss6;

array score{*} z_1-z_6;

do i=**1** to **6**;

if miss{i}<=**4.5**% then do;

do j=i to **6**;

score{j}=**.**;

end;

end;

end;

drop i j;

**run**;

**data** long_full;set _4;

z=z_01; time=-**0.5**;output;

z=z_02; time=-**1**;output;

z=z_03; time=-**1.5**;output;

z=z_04; time=-**2**;output;

z=z_0; time=**0**;output;

z=z_1; time=**0.5**;output;

z=z_2; time=**1**;output;

z=z_3; time=**1.5**;output;

z=z_4; time=**2**;output;

z=z_5; time=**2.5**;output;

z=z_6; time=**3**;output;

keep id group outcome z time;

**run**;

*--------------------Univariate model-------------------;

*==============Outcome 1: Vision acuity-------------------;

**proc** **mixed** data=long_full noclprint=**2**;

where outcome=**1** and **0**<=time<=**3**;

class group(ref='P') id;

model z=group time group*time/s;

random int time/type=un sub=id;

ods output solutionF=sol1;

**run**;

*==============Outcome 1: Thalamus Volume Z-score-------------------;

**proc** **mixed** data=long_full noclprint=**2**;

where outcome=**2** and **0**<=time<=**3**;

class group(ref='P') id;

model z=group time group*time/s;

random int time/type=un sub=id;

ods output solutionF=sol2;

**run**;

*--------------------Multivariate model: 3yr run-in-------------------;

**proc** **nlmixed** data=long_full method=firo;

parms int0 **0.5** int1 -**2.4938** slope00 **0.06796** slope0 **0.06796** slope11 -**0.08184** slope1 -**0.08184** theta **0**

a11=**0.3827** a21=**0.02670** a22=-**0.03932** a31=-**0.05983** a32=**0.02725** a33=**1.0655**

a41=-**0.01696** a42=**0.01017** a43=-**0.03** a44=**0.08**

error1=**0.005322** error2=**0.01079**

;

v11 = a11*a11; v21 = a11*a21; v22 = a21*a21 + a22*a22;

v31=a31*a11;v32=a31*a21+a32*a22;v33=a31****2**+a32****2**+a33****2**;

v41=a41*a11;v42=a41*a21+a42*a22;v43=a41*a31+a42*a32+a43*a33;

v44=a41****2**+a42****2**+a43****2**+a44****2**;

if group='P' and outcome=**1** and time<=**0**

then mu=(int0+u0)+(slope00+u1)*time;

if group='P' and outcome=**1** and time>**0**

then mu=(int0+u0)+(slope0+u1)*time;

if group='T' and outcome=**1** and time<=**0**

then mu=(int0+u0)+(slope00+u1)*time;

if group='T' and outcome=**1** and time>**0**

then mu=(int0+u0)+(slope0*(**1**-theta)+u1)*time;

if group='P' and outcome=**2** and time<=**0**

then mu=(int1+m0)+(slope11+m1)*time;

if group='P' and outcome=**2** and time>**0**

then mu=(int1+m0)+(slope1+m1)*time;

if group='T' and outcome=**2** and time<=**0**

then mu=(int1+m0)+(slope11+m1)*time;

if group='T' and outcome=**2** and time>**0**

then mu=(int1+m0)+(slope1*(**1**-theta)+m1)*time;

if outcome=**1** then error=error1;

if outcome=**2** then error=error2;

model z~normal(mu,error);

random u0 u1 m0 m1~normal([**0**,**0**,**0**,**0**],[v11,v21,v22,v31,v32,v33,v41,v42,v43,v44])

subject=id;

ods output ParameterEstimates =_2RunIn ConvergenceStatus=status2;

**run**;

*--------------------Multivariate model: 3 yrs, No run-in-------------------;

**proc** **nlmixed** data=long_full method=firo;

where **0**<=time<=**3**;

parms int0 **0.5** int1 -**2.4938** slope0 **0.06796** slope1 -**0.08184** theta **0**

a11=**0.3827** a21=**0.02670** a22=-**0.03932** a31=-**0.05983** a32=**0.02725** a33=**1.0655**

a41=-**0.01696** a42=**0.01017** a43=-**0.03** a44=**0.08**

error1=**0.005322** error2=**0.01079**

;

v11 = a11*a11; v21 = a11*a21; v22 = a21*a21 + a22*a22;

v31=a31*a11;v32=a31*a21+a32*a22;v33=a31****2**+a32****2**+a33****2**;

v41=a41*a11;v42=a41*a21+a42*a22;v43=a41*a31+a42*a32+a43*a33;

v44=a41****2**+a42****2**+a43****2**+a44****2**;

if group='P' and outcome=**1** then mu=(int0+u0)+(slope0+u1)*time;

if group='T' and outcome=**1** then mu=(int0+u0)+(slope0*(**1**-theta)+u1)*time;

if group='P' and outcome=**2** then mu=(int1+m0)+(slope1+m1)*time;

if group='T' and outcome=**2** then mu=(int1+m0)+(slope1*(**1**-theta)+m1)*time;

if outcome=**1** then error=error1;

if outcome=**2** then error=error2;

model z~normal(mu,error);

random u0 u1 m0 m1~normal([**0**,**0**,**0**,**0**],[v11,v21,v22,v31,v32,v33,v41,v42,v43,v44])

subject=id;

ods output ParameterEstimates =firo_full3 ConvergenceStatus=status3;

**run**;
